# Neural networks and foundation models: two strategies for EEG-to-fMRI prediction

**DOI:** 10.3389/fsysb.2025.1715692

**Published:** 2025-12-17

**Authors:** Maël Donoso

**Affiliations:** Ouroboros Neurotechnologies, Lausanne, Switzerland

**Keywords:** EEG-to-fMRI prediction, EEG, fMRI, foundation model, neuroimaging, neurofeedback, LLM, chain of thought

## Abstract

Electroencephalography (EEG) and functional Magnetic Resonance Imaging (fMRI) are two widely used neuroimaging techniques, with complementary strengths and weaknesses. Predicting fMRI activity from EEG activity could give us the best of both worlds, and open new horizons for neuroscience research and neurotechnology applications. Here, we formulate this prediction objective both as a classification task (predicting whether the fMRI signal increases or decreases) and a regression task (predicting the value of this signal). We follow two distinct strategies: training classical machine learning and deep learning models (including MLP, CNN, RNN, and transformer) on an EEG-fMRI dataset, or leveraging the capabilities of pre-trained large language models (LLMs) and large multimodal models. We show that predicting fMRI activity from EEG activity is possible for the brain regions defined by the Harvard-Oxford cortical atlas, in the context of subjects performing a neurofeedback task. Interestingly, both strategies yield promising results, possibly highlighting two complementary paths for our prediction objective. Furthermore, a Chain-of-Thought approach demonstrates that LLMs can infer the cognitive functions associated with EEG data, and subsequently predict the fMRI data from these cognitive functions. The natural combination of the two strategies, i.e., fine-tuning an LLM on an EEG-fMRI dataset, is not straightforward and would certainly require further study. These findings could provide important insights for enhancing neural interfaces and advancing toward a multimodal foundation model for neuroscience, integrating EEG, fMRI, and possibly other neuroimaging modalities.

## 1 Introduction

Electroencephalography (EEG) and functional Magnetic Resonance Imaging (fMRI) are two widely used neuroimaging techniques for human brain activity investigation. While EEG provides a direct measure of the electrical activity of the brain by using electrodes placed on the scalp, fMRI measures the activity of the brain indirectly, by detecting changes in the cerebral blood flow and oxygen demand. As a consequence, EEG is typically used to record the activity of cortical areas, whereas fMRI can detect the activity of subcortical regions as well. EEG provides a high temporal resolution, and is a relatively simple and inexpensive neuroimaging technique, compatible with wearable devices. By contrast, fMRI offers a high spatial resolution, at the expense of a greater complexity and cost, and requires the subject to remain immobile inside the scanner. These complementary strengths and weaknesses motivated the emergence of EEG-fMRI research, in which both techniques are used simultaneously (Abreu et al., 2018; Warbrick, 2022).

### 1.1 Predicting fMRI from EEG

The accessibility of EEG-fMRI datasets allowed researchers to explore whether fMRI activity could be predicted from EEG activity. Early research demonstrated that blood oxygenation level dependent (BOLD) (Ogawa et al., 1990) fluctuations in the occipital cortex can be predicted from EEG data, using a linear combination of B-spline basis functions (Sato et al., 2010). Beyond the occipital cortex, two early studies reported the successful prediction of amygdala activity, using classical machine learning models such as Ridge regression (Meir-Hasson et al., 2014; Keynan et al., 2016). Another study focused on connectivity, by applying a model based on sparse canonical correlation analysis to the prediction of the fMRI connectome from the EEG connectome at the scale of the entire brain (Deligianni et al., 2014).

More recently, deep learning models were applied to the prediction of fMRI activity from EEG activity, including convolutional neural networks (Li et al., 2019), attentional graphs (Calhas et al., 2023a), autoencoders and generative adversarial networks (Calhas et al., 2023b). Two preprints proposed novel neural network architectures with the objective to improve interpretability (Kovalev et al., 2022; Semenkov et al., 2024). Research also focused on addressing the hemodynamic delay of the BOLD signal (Simoes et al., 2020), and on predicting the activity of specific brain regions such as the inferior frontal gyrus (Or-Borichev et al., 2023) and the ventral striatum (Singer et al., 2023), respectively associated with cognitive control and reward processes.

Very recently, a series of studies and preprints proposed additional approaches, such as using sinusoidal representation networks (Li et al., 2024a), transformers (Li et al., 2024b; Lanzino et al., 2024), and simpler neural networks inspired by the U-Net architecture (Grover Roos et al., 2025). Overall, current research consistently highlights that predicting fMRI activity from EEG activity could significantly enhance neuroimaging capabilities by giving us the best of both worlds, and open new horizons for neuroscience research and neurotechnology applications (Calhas et al., 2023a; Calhas et al., 2023b; Kovalev et al., 2022; Semenkov et al., 2024; Simoes et al., 2020; Or-Borichev et al., 2023; Singer et al., 2023; Li et al., 2024a; Li et al., 2024b; Lanzino et al., 2024; Grover Roos et al., 2025).

### 1.2 Neurofeedback

One of the neurotechnologies that might benefit the most from EEG-to-fMRI prediction is neurofeedback (NF). NF consists in providing real-time information to a subject about their own brain activity, and encouraging them to adapt their behavior according to this measure. The objective of a NF protocol is for the subject to learn self-regulation and reach a certain cognitive state, whether by increasing or decreasing the power of specific EEG frequency bands (EEG NF), the fMRI activity in specific brain regions (fMRI NF), or a combination of both (EEG-fMRI NF) (Ciccarelli et al., 2023). A study demonstrated that fMRI NF scores and EEG-fMRI NF scores can be predicted from EEG data using a sparse regression model, and that this prediction adds significant information compared to EEG NF scores alone (Cury et al., 2020).

Since the prediction of fMRI activity from EEG activity is an emerging area of research, there is, to our knowledge, no systematic review identifying the most promising brain regions and cognitive processes that could be targeted for EEG-to-fMRI prediction. Our objective is not to provide such a systematic review. However, based on their importance for understanding human cognition, we speculate that the brain regions associated with valuation (Plassmann et al., 2007; Kolling et al., 2012), motivation (Pessiglione et al., 2007; Lebreton et al., 2009), exploration (Boorman et al., 2009), decision-making (Koechlin et al., 2003; Badre et al., 2009; Alexander and Brown, 2011), learning (O’Doherty et al., 2004; Daw et al., 2011), and reasoning (Donoso et al., 2014), in particular, might be valuable candidates. Arguably, all these cognitive functions are engaged during a NF protocol, potentially highlighting the importance of EEG-fMRI NF datasets for future research. This perspective motivated us to select such a dataset for our experiments, although our conclusions do not depend on the fact that the EEG-fMRI data was acquired during a NF protocol.

### 1.3 Toward a multimodal foundation model

We argue that a model capable of predicting fMRI activity from EEG activity at the scale of the entire brain would meet the definition of a foundation model, since it would likely be trained on broad data and could be adapted to a wide range of tasks (Bommasani et al., 2021). Recently, foundation models focusing on a single neuroimaging modality have been developed using self-supervised learning, whether for EEG (Kostas et al., 2021; Jiang et al., 2024a; Cui et al., 2024; Ogg et al., 2024) or fMRI (Shi et al., 2023; Ortega Caro et al., 2023; Ma et al., 2025). However, the model we envision would serve a different purpose. It would be a multimodal foundation model, capable of performing “neural translation” between several neuroimaging modalities. Advancing toward this multimodal foundation model could come with significant challenges, considering both the scarcity and heterogeneity of EEG-fMRI datasets.

Here, we suggest that these challenges might eventually be overcome using a combination of two strategies. The first, classical strategy is the supervised learning approach, which consists in training classical machine learning and deep learning models on an EEG-fMRI dataset. The second, novel strategy consists in directly leveraging the capabilities of pre-trained large language models (LLMs) and large multimodal models (LMMs) for this prediction objective. Indeed, LLMs such as Gemma-2-2B-IT (Riviere et al., 2024) and Llama-3.2-3B-Instruct (Grattafiori et al., 2024), and LMMs such as PaliGemma2-3B-Mix-224 (Steiner et al., 2024) are all pre-trained on extensive corpora of documents, which we assume should include a significant number of neuroscience articles and books, or other sources of knowledge on EEG and fMRI patterns. This extensive pre-training might help us to overcome the scarcity and heterogeneity of EEG-fMRI datasets, by effectively outsourcing a part of the problem. Foundation models are rapidly emerging as powerful instruments for accelerating scientific discovery (Griffin et al., 2024; Gottweis and Natarajan, 2025), including in neuroscience (Wang and Chen, 2024), and a very recent study explored the possibility of encoding EEG signals as LLM-compatible tokens (Jiang et al., 2024b). However, to our knowledge, LLMs and LMMs have not yet been directly leveraged for the complex multimodal task of EEG-to-fMRI prediction.

### 1.4 Two strategies for EEG-to-fMRI prediction

Here, we evaluate these two strategies, i.e., training our own models or relying on pre-trained foundation models, on the same EEG-fMRI dataset. For both strategies, we formulate the prediction objective as a classification task: predicting whether the fMRI signal increases or decreases based on the EEG signal. For the first strategy, i.e., training our own models, we also add a regression task: predicting the value of the fMRI signal based on the EEG signal. Critically, leveraging LLMs or LMMs implies that both features and targets should be designed in order to be described by relevant keywords: EEG frequency bands “with names” instead of arbitrary spectral representations, fMRI brain regions “with names” instead of arbitrary voxel positions, and semantically meaningful classes (“increase”, “decrease”). Therefore, we do not leverage LLMs or LMMs for the regression task, whose targets are numerical.

Since neuroscience articles and books often associate EEG and fMRI patterns with cognitive functions, the keywords describing the latter might serve as intermediate representations between these two neuroimaging modalities. Following this idea, we hypothesize that LLMs could have the capability to infer the cognitive functions associated with EEG data, and subsequently predict the fMRI data from these cognitive functions. We test this hypothesis by implementing a Chain-of-Thought (CoT) (Jason et al., 2022) approach separating these two steps. Finally, we attempt the natural combination of the two strategies by fine-tuning an LLM on an EEG-fMRI dataset, in order to complement its pre-trained capabilities with additional, task-specific knowledge.

While potentially intriguing, the idea that a multimodal foundation model for EEG-to-fMRI prediction might be partially built upon existing language or vision-language foundation models is consistent with several emerging trends in AI research. In particular, it resonates with the growing interest in integrating foundation models with more specific models to address complex multimodal tasks (Brohan et al., 2023; Bansal et al., 2024). Furthermore, it aligns with the idea of using language as a universal interface between different data modalities, a strategy which proved to be successful, in a different field, to predict the evolution of proteins (Hayes et al., 2025). Overall, we believe that evaluating the capabilities of pre-trained foundation models, and comparing them with the performance of classical machine learning and deep learning models, is an important step that could open a new path for EEG-to-fMRI prediction.

## 2 Data

We use the publicly available EEG-fMRI NF dataset *A multi-modal human neuroimaging dataset for data integration: simultaneous EEG and fMRI acquisition during a motor imagery neurofeedback task: XP1* (Lioi et al., 2020), which can be downloaded from OpenNeuro (Poldrack et al., 2013), an open repository for neuroimaging data. This dataset is the first published open-access bimodal NF dataset integrating EEG and fMRI, and was used in the EEG-fMRI NF study mentioned earlier (Cury et al., 2020). The neuroimaging files are stored in Brain Imaging Data Structure (BIDS) format (Gorgolewski et al., 2016), and the dataset is released under the CC0 license. The dataset authors conducted a NF experiment in which 10 subjects (8 male, 2 female, median age = 27) were instructed to use EEG NF scores and fMRI NF scores to perform as well as possible in a motor imagery task (i.e., they needed to execute mentally a movement without any muscle activation). The experiment included six conditions, with alternating rest and task blocks within the conditions.

In our research, we focus on three conditions, which were completed in random order by the different subjects: 1) The eegfmriNF condition, corresponding to bimodal EEG-fMRI NF. 2) The eegNF condition, corresponding to unimodal EEG NF. 3) The fmriNF condition, corresponding to unimodal fMRI NF. For each condition, the dataset includes the raw fMRI data, the raw EEG data, and the EEG data preprocessed by the dataset authors. The fMRI data was acquired with a 3T MRI scanner using echo-planar imaging, with a repetition time of 2 s and a voxel size of 2×2×4 mm^3^. The EEG data was acquired with a 64-channel montage based on the extended 10–20 system, at a sampling rate of 5,000 Hz. The dataset authors subsequently resampled the EEG data to 200 Hz, and applied a low-pass filter at 50 Hz during their preprocessing. The acquisition of EEG data in an fMRI environment is technically complex and can result in high noise (Lioi et al., 2020), making it particularly useful that the dataset authors included the preprocessed EEG data. However, since the preprocessed fMRI data is not included, it is necessary to perform some standard fMRI preprocessing steps before running the experiments.

## 3 Methods

### 3.1 Preprocessing

#### 3.1.1 fMRI preprocessing

We preprocess the raw fMRI data using fMRIPrep (Esteban et al., 2019), a robust preprocessing pipeline which automatically performs a series of standard fMRI preprocessing steps, such as motion correction, coregistration, and spatial normalization. In order to ensure subsequent compatibility with the Harvard-Oxford cortical atlas (Kennedy et al., 2003), we normalize the fMRI data using the MNI152Lin output space. We further preprocess the fMRI data using the NiBabel (Brett et al., 2020) and Nilearn (Alexandre et al., 2014) libraries. For each fMRI scan, we extract the average voxel values of our brain regions of interest, which are the regions defined in the Harvard-Oxford cortical atlas. Within this atlas, we use the maximum probability map with no threshold, assigning each voxel to the region with the highest probability, therefore ensuring full and non-overlapping cortical coverage. We remove a systematic drift of the BOLD signal, which tends to increase during an fMRI session. We also normalize the BOLD signal for each region by subtracting the mean and dividing by the standard deviation, and replace the outliers (STD > 3) with the value of the previous scan. The proportion of outliers is below 1% for every brain region. The fMRIPrep preprocessing is documented in Supplementary README.md, and the details about the additional fMRI preprocessing can be found in Supplementary Notebook 1.

#### 3.1.2 EEG preprocessing

We preprocess the EEG data using the MNE-Python (Gramfort et al., 2013) and YASA (Vallat and Walker, 2021) libraries. We divide the EEG data into 2-second segments aligned with the fMRI scans. For each EEG segment, we compute the band powers for a series of frequency bands of interest: delta (1–4 Hz), theta (4–8 Hz), alpha (8–12 Hz), sigma (12–16 Hz), beta (16–30 Hz), and gamma (30–40 Hz). We normalize the band powers for each channel by subtracting the mean and dividing by the standard deviation, and replace the outliers (STD > 4) with the value of the previous segment. The proportion of outliers is below 0.1% for every frequency band. The details can be found in Supplementary Notebook 2.

#### 3.1.3 Outliers

For both fMRI and EEG data, the replacement of outliers with the value of the previous time point is a simple and conservative approach. It allows for the correction of the limited number of outliers in a way that is consistent across both neuroimaging modalities, while minimizing the risk of introducing artificial temporal patterns. For noisier EEG-fMRI datasets, for example, publicly available datasets that may not have been preprocessed to the same extent by the dataset authors, alternative interpolation techniques could be considered, such as spline or autoregressive interpolation for EEG data, and Kalman filtering or low-rank matrix completion for fMRI data.

#### 3.1.4 Features and targets

For the classification task, the target is a binary label indicating whether the normalized BOLD signal is increasing or decreasing between two successive scans. For the regression task, the target is the normalized BOLD signal at a given scan. For both tasks, unless stated otherwise, the features consist of the normalized band powers computed at a given scan, along with those computed during the 5 preceding scans. This 5-scan sequence corresponds to 10 s, a duration that encompasses the peak of the hemodynamic response function (Gary, 1999), therefore improving our chances of capturing, in the EEG data, a trace of the events that influenced the fMRI data. Since the eegNF condition of one subject is missing, we remove this subject from all the experiments.

### 3.2 Machine learning models

We train a series of classical machine learning models using the Scikit-Learn (Pedregosa et al., 2011) library. For the classification task, we select the following models: logistic regression, k-nearest neighbors (KNN), decision tree (DT), random forest (RF), support vector machine (SVM), and extreme gradient boosting (XGBoost) (Chen and Guestrin, 2016). For the regression task, we select the following models: linear regression, KNN, DT, RF, SVM, and XGBoost. We evaluate the accuracy of the classification task, and the mean absolute error (MAE) of the regression task. For both tasks, to ensure a robust evaluation across sessions, we implement a threefold rotation of the available conditions (eegfmriNF, eegNF, fmriNF) within each subject. In each iteration, one condition serves as the train set and another one as the test set, while the remaining condition is left out. This cross-validation design ensures that every condition is used exactly once for training and once for testing. We also fine-tune the number of neighbors for the KNN model, and the depth of the tree for the DT model. The details can be found in Supplementary Notebooks 3-4, 15-16.

### 3.3 Deep learning models

We train a series of deep learning models using the TensorFlow (Abadi et al., 2016) library. We select the following architectures: multi-layer perceptron (MLP), convolutional neural network (CNN) (LeCun et al., 1998), recurrent neural network (RNN) with gated recurrent units (Cho et al., 2014), and transformer (Vaswani et al., 2017). For the classification task, we use the binary cross-entropy loss function and evaluate the accuracy, whereas for the regression task, we use the mean squared error loss function and evaluate the MAE. For both tasks, to ensure a robust evaluation across sessions, we implement a threefold rotation of the available conditions (eegfmriNF, eegNF, fmriNF) within each subject. In each iteration, one condition serves as the train set, another one as the validation set, and the remaining condition as the test set. This cross-validation design ensures that every condition is used exactly once for training, once for validating, and once for testing, while allowing a symmetrical treatment of the classical machine learning and deep learning models. We train our models on the normalized band powers, except for the CNN model, used as a control, for which we rely directly on the EEG signal without band power extraction. For the CNN model, we use 1D convolutional layers, with the EEG channels defined as the input channels of the network. For all models, we use the Adam (Diederik and Jimmy, 2014) optimizer and the ReLU activation function for hidden layers. The details, including the exact architecture and number of trainable parameters for each model, can be found in Supplementary Notebooks 5–8, 17–20.

### 3.4 Foundation models

#### 3.4.1 Large language models

We leverage two LLMs, Gemma-2-2B-IT and Llama-3.2-3B-Instruct, using the Hugging Face Transformers (Wolf et al., 2020) library. We focus on the classification task and the eegfmriNF condition, and since LLMs require significantly more computational resources than our classical machine learning and deep learning models, we select only a subset of our fMRI scans, band powers, and brain regions of interest. In order to further control the complexity of the prompts, we associate each fMRI brain region with a single EEG channel, which serves as its sole predictor. This association follows an *ad hoc* region-channel mapping, mainly based on electrode proximity to the brain region, and established, perhaps fittingly, with the help of an LLM. We query both models with prompts including a general context, the selected EEG channel, band powers, and brain region, the normalized band power values, and a description of the prediction task to perform. In this experiment and the following ones, we select the model parameters in order to ensure a relative variety of responses, and parse the model outputs by detecting the presence of inflected forms of the keywords “increase” or “decrease” (e.g., “*increases*”, “*increased*”, “*increasing*”) in the generated text. The cases where these keywords are missing, or where both keywords are present, are labeled as invalid predictions. We also ensure that the selected subset of brain regions spans at least a reasonable fraction of the cortex and a variety of cognitive functions.

The prompts follow this pattern: “A human subject participated in a neuroscience experiment where EEG data and fMRI data were recorded simultaneously. The EEG band powers were measured every two seconds at electrode [*selected EEG channel*], with the following results: [*EEG results*]. The fMRI BOLD signal was measured in the [*selected brain region*] during the last two seconds. Given the EEG data, is the fMRI signal in this brain region likely increasing or decreasing during these last two seconds? Base your answer on your general knowledge in neuroscience, EEG research, and fMRI research. Since this is a time series, you might need to take into account the hemodynamic response function (HRF), and the fact that after an event, the fMRI response is delayed compared to the EEG response. Please answer with only one word: Increasing or Decreasing. Just give your best prediction, without any explanation.” The selected EEG channel (e.g., “*Fpz*”) and the selected brain region (e.g., “*Frontal Pole*”) are given in plain text. The EEG results are given as a dictionary-like structure, with each frequency band (e.g., *“Beta (16–30 Hz)”*) associated with the comma-separated values of the different time steps, presented between square brackets. The details, including the region-channel mapping, can be found in Supplementary Notebook 9.

#### 3.4.2 Chain-of-thought and fine-tuning

We conduct two additional experiments with Gemma-2-2B-IT and a single EEG channel. In the first experiment, we implement an intermediate reasoning step using a CoT approach. Specifically, we query the model with a first prompt to infer cognitive functions based on EEG data, then use a second prompt to infer fMRI data based on these cognitive functions. Both prompts also include a general context and a description of the prediction task to perform.

The EEG-to-cognition prompts follow this pattern: “A human subject participated in a neuroscience experiment where EEG data and fMRI data were recorded simultaneously. The EEG band powers were measured every two seconds at electrode [*selected EEG channel*], with the following results: [*EEG results*]. Given the EEG data, which cognitive functions is the subject likely engaging in? Base your answer on your general knowledge in neuroscience and EEG research. Please answer with only a list of cognitive functions. Just give your best prediction, without any explanation.” The subsequent cognition-to-fMRI prompts follow this pattern: “A human subject participated in a neuroscience experiment where EEG data and fMRI data were recorded simultaneously. This subject experienced the following cognitive functions: [*predicted cognitive functions*]. The fMRI BOLD signal was measured in the [*selected brain region*]. Given the cognitive functions, is the fMRI signal in this brain region likely increasing or decreasing? Base your answer on your general knowledge in neuroscience and fMRI research. Please answer with only one word: Increasing or Decreasing. Just give your best prediction, without any explanation.” The predicted cognitive functions (e.g., “*Attention*, *Working Memory*”) are given in plain text. The details can be found in Supplementary Notebook 10.

In the second experiment, we fine-tune the model with parameter-efficient fine-tuning (Dettmers et al., 2023) on input-output pairs obtained from the fmriNF condition, before evaluating again its performance on the eegfmriNF condition. Fine-tuning is implemented by applying low-rank adaptation (Hu et al., 2022) to the query and value projection layers, and the model is optimized using the AdamW (Loshchilov and Hutter, 2017) optimizer. To improve stability and prevent erratic behavior (e.g., the model generating a long chain of *“increase decrease increase decrease…”*), the model is fine-tuned independently for each subject-region pair. We use the same prompt pattern as for the initial, single-step experiment with Gemma-2-2B-IT. The details can be found in Supplementary Notebook 11.

#### 3.4.3 Large multimodal model

We leverage one LMM, PaliGemma2-3B-Mix-224, using the Hugging Face Transformers library. We focus again on the classification task, the eegfmriNF condition, and a subset of our fMRI scans and brain regions of interest. However, instead of using multiple frequency bands and a single EEG channel, we take the opposite approach. Specifically, we create a topographic map using the MNE-Python library, displaying the beta band power (16–30 Hz) across all EEG channels. We prompt the model with this image, along with a general context, the selected brain region, and a description of the prediction task to perform.

The prompts follow this pattern: “A human subject participated in a neuroscience experiment where EEG data and fMRI data were recorded simultaneously. This EEG topographic map shows the brain activity pattern observed for the band power [*selected frequency band*]. The fMRI BOLD signal was measured in the [*selected brain region*] four seconds after that. Given the EEG data, is the fMRI signal in this brain region likely increasing or decreasing? Base your answer on your general knowledge in neuroscience, EEG research, and fMRI research. Please answer with only one word: Increasing or Decreasing. Just give your best prediction, without any explanation.” The selected frequency band (e.g., “*Beta (16–30 Hz*)”) and the selected brain region (e.g., “*Frontal Pole*”) are given in plain text, and the topographic map is added to the prompt. The details can be found in Supplementary Notebook 12.

#### 3.4.4 Large language model with five EEG channels

We conduct one last experiment with Gemma-2-2B-IT, this time using an extended version of the region-channel mapping, where each fMRI brain region is now associated with five EEG channels instead of one. We use essentially the same prompt pattern as for the initial, single-channel experiment with Gemma-2-2B-IT, but adapt the structure of the EEG results to accommodate the new multichannel information. Specifically, the EEG results are presented as a hierarchical structure, where the frequency bands and their corresponding values are given separately for each electrode. The details, including the extended region-channel mapping, can be found in Supplementary Notebook 23.

### 3.5 Statistical tests

For each model, we compare the mean accuracy or MAE to a baseline, across all subject-region pairs (for the classical machine learning and deep learning models: 9 subjects × 49 regions = 441 pairs per cross-validation iteration, totaling 1323 pairs overall). Since we do not assume normality, we perform a one-sided Wilcoxon signed-rank test using the SciPy (Virtanen et al., 2020) library, proceeding similarly for the classification and regression tasks (but searching for opposite effects: higher accuracy for classification, lower MAE for regression). For the foundation models, we also perform McNemar tests, pooling together the predictions from all subjects and brain regions, to compensate for the smaller sample size. Indeed, not only are the foundation models evaluated on a selection of our fMRI scans of interest, but missing or ambiguous predictions must be dynamically excluded, resulting in significantly fewer data points. We also report additional metrics, such as the rank-biserial correlation (RBC), the common language effect size (CLES), as well as the Pearson correlation coefficient for regression, and we perform Wilcoxon signed-rank tests between each pair of models for direct statistical comparison. Wilcoxon signed-rank tests are also performed individually for each region of interest (ROI), corresponding to the cortical regions of the Harvard-Oxford atlas.

For the classical machine learning and deep learning models, the baselines for our statistical tests are relatively straightforward. For the classification task, we use as the baseline the constant prediction of an increase, which corresponds to the majority class, although the two classes are almost perfectly balanced (∼50% each). For the regression task, we use as the baseline the constant prediction of the mean value of the signal, which is zero due to the normalization. For the foundation models, given the lower number of fMRI scans and their more variable target distribution, we proceed differently. We use as the baseline the predictions of a model that randomly samples labels from the true target distribution of the selected fMRI scans, excluding the missing or ambiguous cases from this distribution. We repeat the sampling for 1,000 iterations (Wilcoxon tests) or 10,000 iterations (McNemar tests). The details can be found in Supplementary Notebooks 13, 21, 24, 26.

## 4 Results

### 4.1 Machine learning models

**FIGURE 1:**
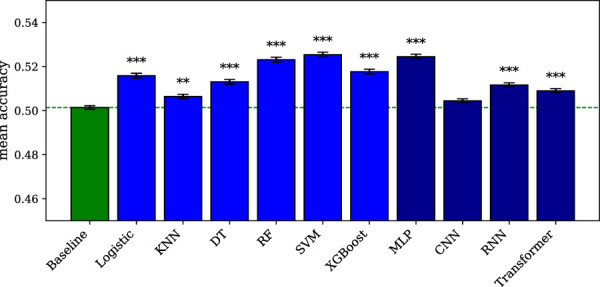
Machine learning and deep learning models for classification. The error bars represent the standard error of the mean across subject-region pairs. Significance is indicated by asterisks: * for p<0.05 , ** for p<0.01 , and *** for p<0.001 . The dashed line indicates the baseline level.

**FIGURE 2:**
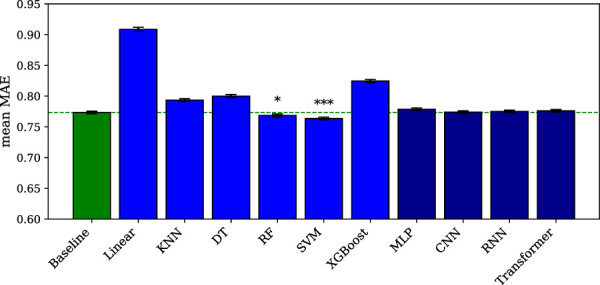
Machine learning and deep learning models for regression. The error bars represent the standard error of the mean across subject-region pairs. Significance is indicated by asterisks: * for p<0.05 , ** for p<0.01 , and *** for p<0.001 . The dashed line indicates the baseline level.

All the classical machine learning models for classification, i.e., the logistic regression, KNN, DT, RF, SVM, and XGBoost models, reach an accuracy higher than the baseline (p < 0.01 for KNN, p < 0.001 for the other models), as shown in Figure 1. The best model (SVM) performs slightly better (p = 0.033) than the second best (RF). Among the classical machine learning models for regression, only the RF (p < 0.05, Pearson r = 0.145) and SVM (p < 0.001, Pearson r = 0.175) models perform better than the baseline, as shown in Figure 2. The detailed results can be found in Supplementary Notebooks 14, 22, and the statistics in Supplementary Tables A–D.

### 4.2 Deep learning models

Among the deep learning models for classification, the MLP, RNN, and transformer models reach an accuracy higher than the baseline (p < 0.001), as shown in Figure 1. However, the training graphs of all models except MLP show signs of overfitting, with the validation loss often stagnating or even increasing over the epochs. The best classical machine learning model (SVM) reaches a slightly better performance than the best deep learning model (MLP), but the difference is not statistically significant (p = 0.144). In return, the MLP model outperforms the second best classical machine learning model (RF), but again, the difference remains below the significance threshold (p = 0.181). Among the deep learning models for regression, no model performs better than the baseline, as shown in Figure 2. We also evaluate the number of floating-point operations (FLOPs) necessary for running the MLP (∼23M), CNN (∼51M), RNN (∼39M), and transformer (∼76M) models, to confirm that the higher performance of the MLP model for the classification task is not due to model complexity. The training graphs are displayed in Supplementary Notebooks 5–8, 17–20, while the detailed results can be found in Supplementary Notebooks 14, 22, and the statistics in Supplementary Tables A–D.

### 4.3 Foundation models

Neither the LLMs nor the LMM perform better than the baseline when the predictions are evaluated using the one-sided Wilcoxon signed-rank test. However, when the predictions are evaluated using the less conservative McNemar test across multiple iterations, a more nuanced view emerges. For the different instances of the Gemma model prompted with a single EEG channel, we observe a relatively low median p-value (median p = 0.12, 0.16, 0.15, 0.14) and a high proportion of statistically significant iterations (proportion p < 0.05 = 29%, 23%, 23%, 24%). The Llama model and the Gemma model prompted with five EEG channels show the opposite trend, with a relatively high median p-value (median p = 0.66, 0.64) and a low proportion of statistically significant iterations (proportion p < 0.05 = 0.5%, 0.7%). The PaliGemma model shows an intermediate pattern (median p = 0.31, proportion p < 0.05 = 8.6%). The proportion of missing or ambiguous predictions varies across models, but is always less than 10%. The detailed results can be found in Supplementary Notebooks 13–14, 24–25, and the statistics in Supplementary Tables E, F.

**FIGURE 3:**
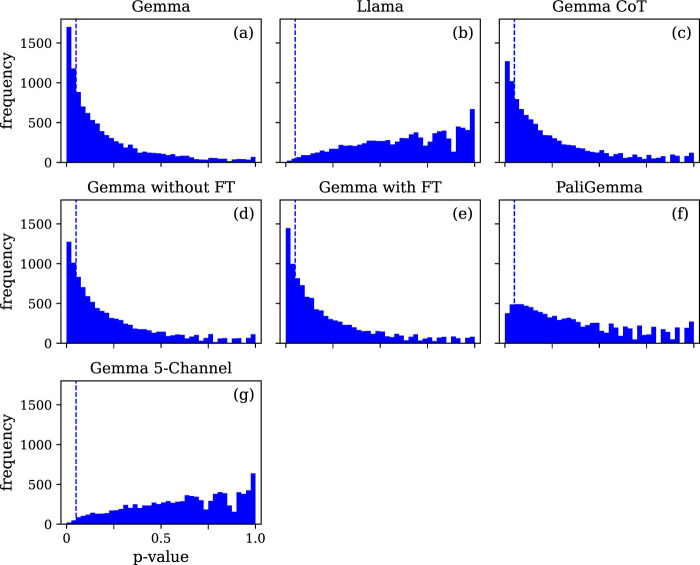
Distribution of p-values for the Gemma **(a)**, Llama **(b)**, Gemma CoT **(c)**, Gemma without fine-tuning **(d)**, Gemma with fine-tuning **(e)**, PaliGemma **(f)**, and Gemma 5-channel **(g)** foundation model instances. The dashed lines indicate the p<0.05 threshold.

The visualization of the p-value distributions using histograms provides important insights as well. As shown in Figure 3, for the different instances of the Gemma model prompted with a single EEG channel, we observe a right-skewed p-value distribution, with a mode near p = 0 and a long tail toward p = 1, suggesting that the Gemma model achieves small but reliable gains over the baseline, whether it is used with direct prompting or with a CoT approach. The Llama model and the Gemma model prompted with five EEG channels show almost the opposite trend, with a weakly left-skewed p-value distribution, suggesting an absence of improvement over the baseline. The PaliGemma model shows again an intermediate pattern, with a weakly right-skewed p-value distribution, suggesting a more ambiguous behavior. We also evaluate the number of FLOPs necessary for running the Gemma single-channel (∼3T), Llama (∼3.7T), PaliGemma (∼1.6T), and Gemma five-channel (∼6.7T) model instances, obtaining values that stand several orders of magnitude above those measured for the classical machine learning and deep learning models. Comparing the performance of the Gemma model with and without fine-tuning, we observe that the difference is not statistically significant, whether the comparison is performed using the one-sided Wilcoxon signed-rank test (p = 0.42) or the McNemar test (p = 0.87). By contrast, the Gemma model prompted with a single EEG channel performs significantly better than the same model prompted with five EEG channels, whether the comparison is performed using the one-sided Wilcoxon signed-rank test (p = 0.018) or the McNemar test (p = 0.021).

### 4.4 Regions of interest

**FIGURE 4:**
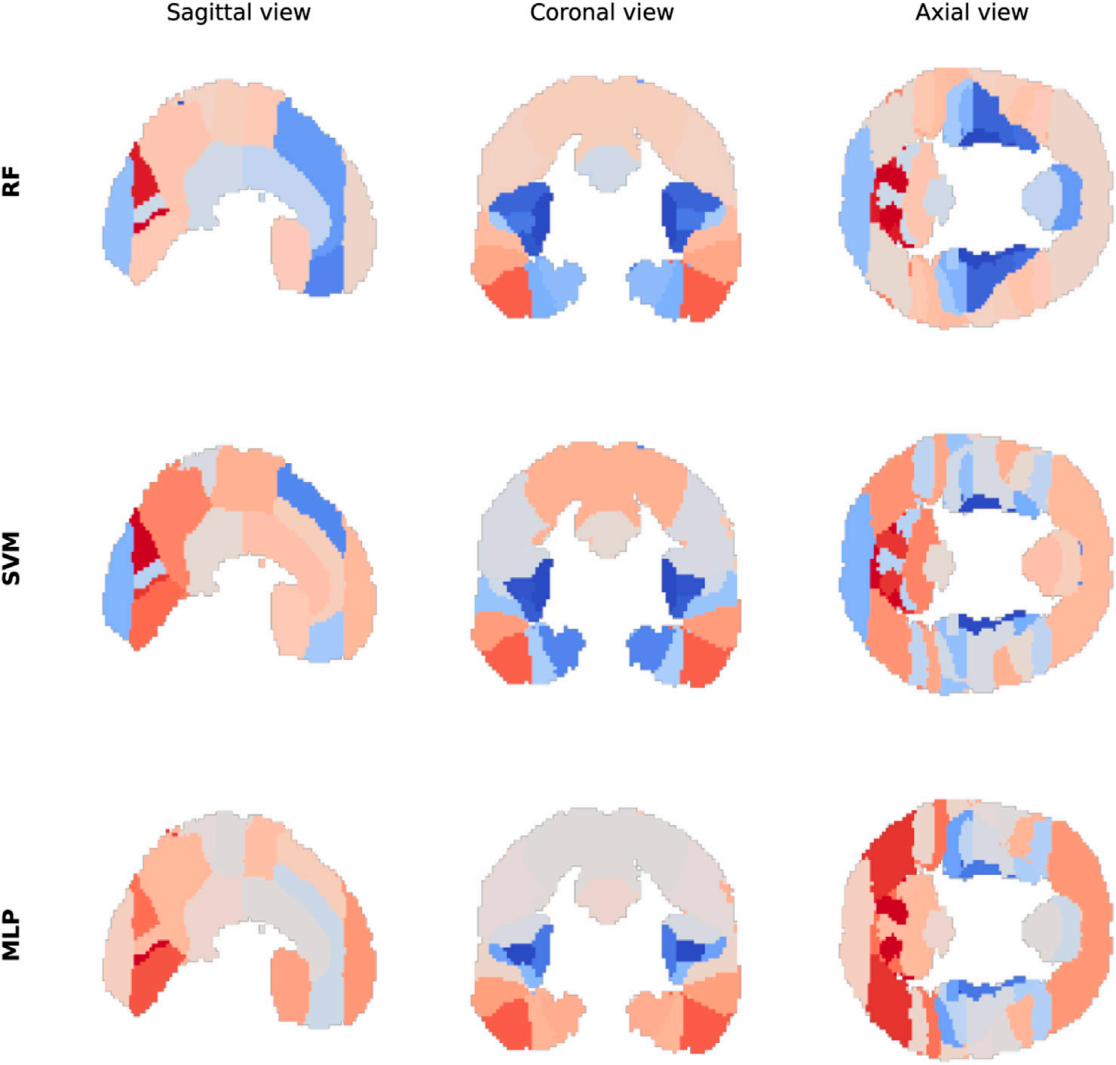
Distribution of accuracy values across cortical regions. The ROI accuracy values are displayed for the three best-performing classification models (RF, SVM, MLP) across the sagittal, coronal, and axial views. Blue indicates lower accuracy, and red indicates higher accuracy.

The region-wise analyses reveal substantial variability in the performance of the models across the cortical regions of the Harvard-Oxford atlas. For both the classification and the regression tasks, the classical machine learning and deep learning models reach their best performance in ROIs such as the cuneal cortex, the posterior division of the inferior temporal gyrus, and the inferior division of the lateral occipital cortex. By contrast, the performance of the models is more limited in ROIs such as the insular cortex and Heschl’s gyrus. Focusing on the classification task and the RF, SVM, and MLP models, the spatial distribution of the accuracy reveals some interesting patterns, as shown in Figure 4. While the three models display distinct local differences, they also share some global similarities, such as a lower accuracy around the insular cortex, and a higher accuracy in some parts of the occipital cortex. The detailed results can be found in Supplementary Notebooks 26–27, and the statistics in Supplementary Tables G-J.

## 5 Discussion

### 5.1 First strategy

The first strategy, which consists in training classical machine learning and deep learning models, proves to be the most successful. Interestingly, the MLP model is one of the best-performing models for the classification task, and the only deep learning model achieving similar or higher accuracy than the classical machine learning models. The superiority of the MLP model over the CNN, RNN, and transformer models is not due to model complexity, as confirmed by the number of FLOPs. Rather, it suggests that the relevant patterns for EEG-to-fMRI prediction might be globally distributed across space (EEG channels) and time (fMRI scans), therefore favoring feedforward neural network architectures which do not rely too strongly on spatial or temporal priors. We might expect that this global distribution of relevant features could also favor text-only LLMs over LMMs, since the latter typically rely on data with stronger structure, such as images. The superiority of the MLP model is also consistent with a very recent preprint suggesting that simple neural networks for EEG-to-fMRI prediction can outperform more complex ones (Grover Roos et al., 2025).

The region-wise analyses are constrained by the objective of this research, which is to evaluate the two strategies for EEG-to-fMRI prediction in a relatively symmetrical manner. While the use of fMRI brain regions “with names”, such as the cortical regions of the Harvard-Oxford atlas, is necessary for leveraging LLMs and LMMs, it is unclear whether these ROIs would be optimal in the context of classical machine learning and deep learning models alone. For example, the relatively large “Occipital Pole” region, observable at the far left of the sagittal view in Figure 4, displays limited accuracy for the RF and SVM models, whereas neighboring occipital areas show higher predictability. This suggests that the size of the ROIs, along with other variables, might have an impact on the quality of the prediction, possibly because larger regions tend to implement more diverse cognitive processes. We speculate that training the models on more specific brain regions, or even directly at the level of individual voxels, could potentially provide additional insights on the spatial variations in predictability, at the expense of a greater computational cost. Despite this general constraint, the patterns observed seem informative for evaluating the possibilities of EEG-to-fMRI prediction, while remaining consistent with our scientific knowledge on the human brain. In particular, the low accuracy observed around the insular cortex may reflect the difficulty of measuring the electrical activity of this deep cortical region using EEG, whereas the high accuracy observed in some parts of the occipital cortex may be explained by the strong EEG correlates of visual processes.

Intriguingly, although the majority of our neural network architectures achieve significant results in the classification task, none of them performs better than the baseline in the regression task, whereas the RF and SVM models do. This limitation of our neural networks should not be interpreted as evidence that deep learning models are generally unable to perform such regression task: although EEG and fMRI remain very different measures of the activity of the brain, recent studies have demonstrated the feasibility of the EEG-to-fMRI regression task when using more complex and specialized deep learning models, in particular in resting-state contexts (Li et al., 2024b). Rather, this limitation of our neural networks seems to indicate that our simple architectures are insufficient to extract meaningful information for the regression task, despite their success on the classification task. In other words, the complexity threshold for a minimally working neural network might be higher for the regression task, possibly because the fluctuations of EEG or fMRI data at multiple time scales prevent the models from precisely inferring the amplitude of the fMRI signal, while still allowing them to infer its direction. Remarkably, the RF and SVM models maintain a better performance, suggesting that these simple machine learning models might be less sensitive to noise and drift in this particular context. The extent to which EEG data can be used to predict the amplitude of fMRI data is an open question, and the level of precision required for real-world applications will most certainly depend on the intended usage. Nevertheless, the differences observed between the classification and regression tasks suggest that targeting the direction of the fMRI signal, rather than its amplitude, might provide an easier path forward for enhancing neural interfaces with EEG-to-fMRI prediction, particularly in the context of wearable devices with limited computing power.

### 5.2 Second strategy

The second strategy, which consists in directly leveraging the capabilities of pre-trained foundation models, shows promising results as well. Although the observed effects are much smaller than for the classical machine learning and deep learning models, the Gemma model prompted with a single EEG channel achieves statistically reliable gains over the baseline. Furthermore, our CoT approach demonstrates that the Gemma model can infer the cognitive functions associated with EEG data, and subsequently predict the fMRI data from these cognitive functions. This suggests the possibility that the performance of the Gemma model with direct prompting might be driven by a similar mechanism, with the model implicitly relying on cognitive functions as an intermediate reasoning step between EEG and fMRI, even in the absence of native CoT capabilities. The lower performance of the Llama and PaliGemma models might reflect their intrinsic limitation for this particular prediction task, or simply suggest that more exploration is needed to adapt the prompts and parameters for each foundation model. The absence of improvement observed after fine-tuning the Gemma model may seem intriguing, but while it is difficult to provide a definitive interpretation, a very recent study highlighted that the result of fine-tuning an LLM for a scientific problem depends both on the dataset and on the complexity of the question (Van Herc et al., 2025). This research reported a weak predictive power for complex input variables and a very low number of training data points, which is precisely our situation. Overcoming this difficulty, and establishing a more successful fine-tuning strategy, would certainly require further study.

Another result that may seem counterintuitive is the degradation of the performance of the Gemma model when prompted with five EEG channels instead of one. Classical machine learning and deep learning models would typically benefit from multichannel integration, as adding new EEG channels would allow these models to exploit several complementary sources of information, as well as the interactions between them. Such integration is particularly important in EEG research for decoding brain states and understanding cognitive functions. However, current LLMs are not specifically designed to process complex, structured numerical inputs with the same efficiency. As a result, while the five-channel prompts are substantially longer, requiring more than twice the FLOPs of the single-channel prompts, it is unclear whether this additional information could be reliably leveraged by the model for its task. In particular, the mechanisms by which an LLM would resolve conflicting EEG channels, or channel-specific noise in band power values, are not obvious. Rather, we speculate that the Gemma model might show a better performance in the single-channel case precisely because the limited, focused information allows this model to rely on simple heuristics, extracted from the neuroscience literature and related sources. Longer prompts featuring nested numerical data might degrade these heuristics, resulting in less accurate inferences. Still, whereas current LLMs may be limited in their capacity to integrate complex numerical inputs, future foundation models with more advanced reasoning capabilities might achieve a significantly better performance in such tasks, expanding their utility for EEG-to-fMRI prediction.

### 5.3 Limitations

The EEG-fMRI dataset on which this research is based is limited in size, and unbalanced in terms of sex and age. Given the limited training set, the classical machine learning and deep learning models are evaluated across sessions, but not across subjects. The exact data on which the foundation models have been pre-trained, and in particular the number of neuroscience articles and books, or other sources of knowledge on EEG and fMRI patterns, is not publicly available. Running the foundation models requires significantly more computational resources than running the classical machine learning and deep learning models, which forces us to focus on a subset of our fMRI scans, band powers, and brain regions of interest, and to use an *ad hoc* region-channel mapping. Also because of this computational cost, we only evaluate small foundation models, which may be less performant than larger ones. In general, this research focuses on demonstrating the feasibility of the two strategies for EEG-to-fMRI prediction, and does not aim to achieve the best performance possible.

### 5.4 Future directions

For the first strategy, a natural path forward would be to explore additional preprocessing pipelines and neural network architectures, and to experiment with more specific models, relying more strongly on our knowledge of the human brain. We would certainly benefit from larger EEG-fMRI datasets, and more balanced in terms of sex and age, if they become available in the future. Since the scarcity and heterogeneity of EEG-fMRI datasets is currently a significant obstacle, developing methods for integrating heterogeneous datasets (e.g., different EEG montages, fMRI scan durations, etc.) could also be highly valuable. For the second strategy, the possible future directions are relatively symmetrical. It would make sense to explore additional prompt engineering techniques and model parameters, and to experiment with foundation models specifically pre-trained on the neuroscience literature. We would certainly benefit from more advanced and general foundation models, which will likely become available in the future, in particular if these models have native CoT capabilities, potentially allowing them to reliably leverage multichannel information, and to explicitly use cognitive functions as an intermediate reasoning step between EEG and fMRI. Developing methods for successfully integrating the two strategies, for example by fine-tuning foundation models on existing EEG-fMRI datasets, could also be a promising path. Finally, both strategies could be extended beyond EEG and fMRI, to other neuroimaging modalities such as magnetoencephalography, bringing us a step closer to a multimodal foundation model for neuroscience.

## 6 Conclusion

In this research, we demonstrate the feasibility of predicting fMRI activity from EEG activity by following two distinct strategies: training classical machine learning and deep learning models on an EEG-fMRI dataset, or leveraging the capabilities of pre-trained foundation models. When this prediction objective is formulated as a classification task, the RF, SVM, and MLP models stand out as particularly effective, while the Gemma model prompted with a single EEG channel achieves statistically reliable gains over the baseline, whether it is used with direct prompting or with a CoT approach. The latter case demonstrates that the Gemma model can infer the cognitive functions associated with EEG data, and subsequently predict the fMRI data from these cognitive functions. Although the observed effects are much smaller for the foundation models than for the classical machine learning and deep learning models, the possibility to leverage LLMs and LMMs for this task, and potentially to integrate the two strategies by fine-tuning foundation models on existing EEG-fMRI datasets, could open new horizons for EEG-to-fMRI prediction. As more advanced and general LLMs and LMMs continue to be developed, these models could become increasingly important tools for enhancing neural interfaces and advancing toward a multimodal foundation model for neuroscience.

### 6.1 Reproducibility

The experiments in this research are based on the publicly available EEG-fMRI NF dataset *A multi-modal human neuroimaging dataset for data integration: simultaneous EEG and fMRI acquisition during a motor imagery neurofeedback task: XP1*, released under the CC0 license and accessible via OpenNeuro at https://openneuro.org/datasets/ds002336/versions/2.0.2. The Gemma, Llama, and PaliGemma models are also publicly available. The code is provided in fully commented Jupyter Notebooks (Supplementary Notebooks 1–27), designed to be run in a Conda environment specified by Supplementary ENV.yml. The fMRI preprocessing using fMRIPrep is documented in Supplementary README.md, and must be completed before running the experiments. On a standard personal computer (e.g., MacBook Pro), the fMRIPrep preprocessing takes around 24–36 h, while the full execution of the code requires an additional 8–12 h.

### 6.2 Societal impact

This research does not introduce a new deployable system or asset, and is not expected to have an immediate societal impact. However, by contributing to the long-term objective of EEG-to-fMRI prediction, it could eventually support the development of enhanced neural interfaces, designed to achieve near-fMRI precision while retaining the affordability and wearability of EEG devices. While such neural interfaces remain hypothetical, the possibility to monitor cognitive processes at scale could have a profound impact in many domains, and would naturally require careful reflection and evaluation, considering the sensitive nature of human brain data.

## Data Availability

Publicly available datasets were analyzed in this study. This data can be found here: https://openneuro.org/datasets/ds002336/versions/2.0.2.
