# Supplementary material for: Neural networks and foundation models: two strategies for EEG-to-fMRI prediction
Source: Front Syst Biol. 2025 Dec 17;5:1715692. doi: 10.3389/fsysb.2025.1715692 (PMC12753390; doi:10.3389/fsysb.2025.1715692)
Supplement: Supplementary file 1 [file Supplementaryfile1.zip › Supplementary_Material/Supplementary_Tables.pdf]

TABLE A: Statistics for classification models (Wilcoxon tests)

Results are given with their standard deviation and a 95% confidence interval.

	N	model accuracy	baseline accuracy	p-value	confidence interval	RBC	CLES
**Logistic**	1323	0.516 ± 0.041	0.501 ± 0.03	1.51e-21	0.514 - 0.518	0.306	0.611
**KNN**	1323	0.506 ± 0.036	0.501 ± 0.03	6.51e-03	0.504 - 0.508	0.08	0.536
**DT**	1323	0.513 ± 0.039	0.501 ± 0.03	2.69e-13	0.511 - 0.515	0.24	0.579
**RF**	1323	0.523 ± 0.042	0.501 ± 0.03	1.55e-42	0.521 - 0.525	0.442	0.658
**SVM**	1323	0.525 ± 0.041	0.501 ± 0.03	1.17e-52	0.523 - 0.528	0.502	0.676
**XGB**	1323	0.518 ± 0.041	0.501 ± 0.03	3.69e-26	0.515 - 0.52	0.339	0.619
**MLP**	1323	0.524 ± 0.042	0.501 ± 0.03	1.84e-47	0.522 - 0.527	0.47	0.663
**CNN**	1323	0.504 ± 0.033	0.501 ± 0.03	2.09e-01	0.503 - 0.506	0.028	0.515
**RNN**	1323	0.512 ± 0.035	0.501 ± 0.03	4.34e-13	0.51 - 0.514	0.242	0.575
**Transformer**	1323	0.509 ± 0.036	0.501 ± 0.03	1.91e-06	0.507 - 0.511	0.154	0.549

TABLE B: Statistics for regression models (Wilcoxon tests)

Results are given with their standard deviation and a 95% confidence interval.

	N	model MAE	baseline MAE	p-value	confidence interval	RBC	CLES	Pearson r
**Linear**	1323	0.909 ± 0.128	0.773 ± 0.077	1.00e+00	0.901 - 0.915	-0.85	0.168	0.147
**KNN**	1323	0.793 ± 0.087	0.773 ± 0.077	1.00e+00	0.789 - 0.798	-0.437	0.427	0.09
**DT**	1323	0.8 ± 0.092	0.773 ± 0.077	1.00e+00	0.795 - 0.805	-0.641	0.396	0.067
**RF**	1323	0.769 ± 0.085	0.773 ± 0.077	1.25e-02	0.764 - 0.773	0.071	0.545	0.145
**SVM**	1323	0.764 ± 0.082	0.773 ± 0.077	5.27e-09	0.759 - 0.768	0.182	0.562	0.175
**XGB**	1323	0.825 ± 0.09	0.773 ± 0.077	1.00e+00	0.82 - 0.829	-0.75	0.29	0.114
**MLP**	1323	0.779 ± 0.079	0.773 ± 0.077	1.00e+00	0.774 - 0.783	-0.278	0.477	0.035
**CNN**	1323	0.774 ± 0.076	0.773 ± 0.077	9.56e-01	0.77 - 0.778	-0.054	0.499	0.043
**RNN**	1323	0.775 ± 0.076	0.773 ± 0.077	1.00e+00	0.771 - 0.779	-0.406	0.494	0.012
**Transformer**	1323	0.776 ± 0.078	0.773 ± 0.077	1.00e+00	0.772 - 0.78	-0.119	0.502	0.094

TABLE C: Comparison of classification models (p-values)

Each cell reports the p-value for comparing the model in the row against the model in the column.

	Logistic	KNN	DT	RF	SVM	XGB	MLP	CNN	RNN	Transformer
**Logistic**	-	1.08e-11	3.30e-02	1.00e+00	1.00e+00	8.79e-01	1.00e+00	1.21e-15	1.55e-03	6.17e-07
**KNN**	1.00e+00	-	1.00e+00	1.00e+00	1.00e+00	1.00e+00	1.00e+00	2.24e-02	1.00e+00	9.67e-01
**DT**	9.67e-01	4.58e-06	-	1.00e+00	1.00e+00	1.00e+00	1.00e+00	1.53e-10	1.13e-01	6.59e-04
**RF**	2.12e-08	4.44e-31	1.16e-14	-	9.67e-01	1.22e-05	8.19e-01	1.39e-38	5.50e-18	6.55e-24
**SVM**	1.60e-17	5.38e-41	8.81e-21	3.30e-02	-	2.84e-12	1.44e-01	8.19e-49	3.21e-26	2.50e-31
**XGB**	1.21e-01	2.30e-15	9.76e-05	1.00e+00	1.00e+00	-	1.00e+00	1.37e-19	5.94e-06	5.05e-10
**MLP**	9.42e-13	5.11e-33	6.87e-16	1.81e-01	8.56e-01	1.04e-07	-	1.65e-38	1.83e-20	1.45e-26
**CNN**	1.00e+00	9.78e-01	1.00e+00	1.00e+00	1.00e+00	1.00e+00	1.00e+00	-	1.00e+00	1.00e+00
**RNN**	9.98e-01	2.83e-04	8.87e-01	1.00e+00	1.00e+00	1.00e+00	1.00e+00	7.18e-10	-	1.83e-02
**Transformer**	1.00e+00	3.33e-02	9.99e-01	1.00e+00	1.00e+00	1.00e+00	1.00e+00	3.35e-04	9.82e-01	-

TABLE D: Comparison of regression models (p-values)

Each cell reports the p-value for comparing the model in the row against the model in the column.

	Linear	KNN	DT	RF	SVM	XGB	MLP	CNN	RNN	Transformer
**Linear**	-	1.00e+00	1.00e+00	1.00e+00	1.00e+00	1.00e+00	1.00e+00	1.00e+00	1.00e+00	1.00e+00
**KNN**	2.25e-149	-	1.51e-05	1.00e+00	1.00e+00	2.65e-69	1.00e+00	1.00e+00	1.00e+00	1.00e+00
**DT**	7.36e-122	1.00e+00	-	1.00e+00	1.00e+00	4.21e-49	1.00e+00	1.00e+00	1.00e+00	1.00e+00
**RF**	1.80e-175	3.23e-53	6.90e-117	-	1.00e+00	6.50e-193	1.81e-11	1.38e-03	3.63e-05	4.80e-10
**SVM**	3.40e-199	2.99e-110	2.18e-76	7.72e-05	-	2.32e-164	1.06e-24	1.38e-10	3.29e-13	8.90e-21
**XGB**	3.58e-113	1.00e+00	1.00e+00	1.00e+00	1.00e+00	-	1.00e+00	1.00e+00	1.00e+00	1.00e+00
**MLP**	1.83e-153	2.63e-22	2.08e-49	1.00e+00	1.00e+00	1.93e-111	-	1.00e+00	1.00e+00	9.07e-01
**CNN**	4.73e-158	2.27e-39	1.82e-84	9.99e-01	1.00e+00	1.26e-123	7.19e-13	-	3.22e-14	9.63e-04
**RNN**	3.40e-157	8.63e-36	1.77e-77	1.00e+00	1.00e+00	2.23e-120	1.87e-08	1.00e+00	-	4.52e-02
**Transformer**	5.03e-175	1.03e-24	1.59e-43	1.00e+00	1.00e+00	1.17e-118	9.29e-02	9.99e-01	9.55e-01	-

TABLE E: Statistics for foundation models (Wilcoxon tests)

Results are given with their standard deviation and a 95% confidence interval.

	N	model accuracy	baseline accuracy	p-value	confidence interval	RBC	CLES
**Gemma**	72	0.534 ± 0.106	0.528 ± 0.038	1.82e-01	0.509 - 0.558	0.123	0.555
**Llama**	72	0.51 ± 0.129	0.531 ± 0.037	8.57e-01	0.479 - 0.539	-0.145	0.456
**Gemma CoT**	45	0.545 ± 0.117	0.533 ± 0.041	1.88e-01	0.51 - 0.579	0.154	0.573
**Gemma without FT**	45	0.545 ± 0.116	0.533 ± 0.039	1.42e-01	0.512 - 0.579	0.184	0.555
**Gemma with FT**	45	0.547 ± 0.133	0.534 ± 0.046	1.36e-01	0.508 - 0.585	0.19	0.549
**PaliGemma**	72	0.477 ± 0.142	0.584 ± 0.12	1.00e+00	0.445 - 0.509	-0.54	0.31
**Gemma 5-Channel**	72	0.503 ± 0.118	0.53 ± 0.041	9.67e-01	0.476 - 0.53	-0.25	0.387

TABLE F: Statistics for foundation models (McNemar's tests)

Combined p-values are obtained using Fisher's method. A combined p-value close to 0 suggests an effect, and a combined p-value close to 1 an absence of effect.

	N	model accuracy	baseline accuracy	combined p-value	median p-value	proportion p-value < 0.05
**Gemma**	1386	0.535	0.505	0.00e+00	1.21e-01	0.288
**Llama**	1326	0.508	0.505	1.00e+00	6.61e-01	0.005
**Gemma CoT**	858	0.544	0.51	0.00e+00	1.55e-01	0.229
**Gemma without FT**	870	0.545	0.511	0.00e+00	1.54e-01	0.228
**Gemma with FT**	867	0.547	0.512	0.00e+00	1.44e-01	0.244
**PaliGemma**	670	0.479	0.509	0.00e+00	3.08e-01	0.086
**Gemma 5-Channel**	1327	0.501	0.506	1.00e+00	6.39e-01	0.007

TABLE G: ROI statistics for classification models (RBC values)

The "Mean Region" column summarizes the predictability for a given ROI by averaging the RBC values of the different models. The ROIs are sorted in descending order based on this column, so that the ROIs with the highest general predictability appear first.

	Logistic	KNN	DT	RF	SVM	XGB	MLP	CNN	RNN	Transformer	MEAN REGION
**Cuneal Cortex**	0.883	0.806	0.606	0.897	1	0.974	0.735	0.607	0.877	0.62	**0.801**
**Intracalcarine Cortex**	0.915	0.638	0.687	0.923	0.934	0.81	0.951	0.565	0.567	0.577	**0.757**
**Inferior Temporal Gyrus, posterior division**	0.646	0.587	0.766	0.797	0.854	0.6	0.786	0.681	0.891	0.729	**0.734**
**Lateral Occipital Cortex, inferior division**	0.701	0.714	0.571	0.745	0.727	0.519	0.875	0.233	0.61	0.542	**0.624**
**Subcallosal Cortex**	0.812	0.535	0.703	0.534	0.557	0.598	0.578	0.601	0.686	0.511	**0.612**
**Inferior Temporal Gyrus, temporooccipital part**	0.622	0.556	0.348	0.733	0.738	0.741	0.613	0.446	0.595	0.433	**0.582**
**Middle Temporal Gyrus, posterior division**	0.492	0.647	0.381	0.652	0.714	0.51	0.593	0.286	0.429	0.579	**0.528**
**Occipital Fusiform Gyrus**	0.606	0.425	0.372	0.405	0.84	0.577	0.812	-0.14	0.696	0.513	**0.51**
**Lateral Occipital Cortex, superior division**	0.64	0.249	0.554	0.48	0.733	0.587	0.877	0.102	0.403	0.179	**0.481**
**Lingual Gyrus**	0.537	0.283	0.252	0.538	0.833	0.529	0.8	-0.026	0.442	0.394	**0.458**
**Middle Temporal Gyrus, temporooccipital part**	0.348	0.391	0.125	0.633	0.764	0.575	0.696	0.178	0.363	0.473	**0.455**
**Inferior Temporal Gyrus, anterior division**	0.393	0.425	0.462	0.416	0.423	0.653	0.597	0.398	0.199	0.526	**0.449**
**Temporal Occipital Fusiform Cortex**	0.256	0.094	0.187	0.598	0.565	0.28	0.852	-0.274	0.291	0.399	**0.325**
**Supracalcarine Cortex**	0.5	0.41	-0.134	0.41	0.338	0.209	0.476	0.357	0.209	0.42	**0.32**
**Frontal Medial Cortex**	0.299	0.228	0.21	0.105	0.282	0.547	0.218	0.271	0.68	0.273	**0.311**

**Precuneous Cortex**	0.457	0.047	0.222	0.557	0.772	0.5	0.497	0.02	-0.062	0.07	**0.308**
**Supramarginal Gyrus, anterior division**	0.612	-0.363	0.017	0.499	0.56	0.606	0.52	-0.406	0.45	0.263	**0.276**
**Juxtapositional Lobule Cortex (formerly Supplementary Motor Cortex)**	0.413	-0.16	0.413	0.54	0.647	0.462	0.463	-0.462	0.357	0.062	**0.274**
**Frontal Orbital Cortex**	0.325	0.439	0.237	0.426	0.399	0.249	0.31	0.031	0.283	0.027	**0.273**
**Supramarginal Gyrus, posterior division**	0.19	-0.27	0.172	0.585	0.677	0.369	0.726	-0.507	0.484	0.19	**0.262**
**Superior Temporal Gyrus, posterior division**	0.098	-0.19	0.138	0.593	0.258	0.27	0.344	0.199	0.368	0.435	**0.251**
**Temporal Fusiform Cortex, posterior division**	0.077	0.197	0.203	0.217	0.302	0.274	0.513	0.153	0.255	0.19	**0.238**
**Middle Frontal Gyrus**	0.342	-0.265	0.204	0.649	0.452	0.317	0.532	-0.2	0.141	0.197	**0.237**
**Frontal Pole**	0.259	-0.083	0.31	0.493	0.62	0.222	0.613	-0.453	0.007	-0.157	**0.183**
**Inferior Frontal Gyrus, pars opercularis**	-0.003	-0.209	-0.087	0.564	0.481	0.045	0.513	-0.197	0.303	0.345	**0.176**
**Cingulate Gyrus, anterior division**	0.312	0.145	-0.042	0.38	0.593	0.2	0.291	-0.407	0.117	0.098	**0.169**
**Precentral Gyrus**	0.228	-0.177	-0.13	0.524	0.65	0.267	0.286	-0.153	-0.092	0.16	**0.156**
**Parahippocampal Gyrus, anterior division**	0.153	-0.126	0.173	0.257	0.047	-0.016	0.54	0.15	0.207	0.095	**0.148**
**Parahippocampal Gyrus, posterior division**	0.28	0.095	0.12	0.098	0.211	0.074	0.308	-0.043	0.107	0.182	**0.143**
**Inferior Frontal Gyrus, pars triangularis**	-0.009	-0.116	0.42	0.537	0.353	-0.018	0.125	-0.188	0.15	0.06	**0.131**
**Postcentral Gyrus**	0.248	0.117	0.16	0.511	0.427	0.169	0.305	-0.147	-0.21	-0.307	**0.127**
**Central Opercular Cortex**	0.437	-0.353	-0.148	0.037	0.419	0.345	0.259	-0.163	0.14	0.138	**0.111**
**Temporal Pole**	0.204	-0.182	0.075	0.605	0.661	0.058	0.185	-0.372	0.008	-0.285	**0.096**
**Planum Temporale**	0.212	-0.205	0.199	0.311	0.406	0.098	-0.108	0.316	-0.237	-0.039	**0.095**
**Superior Temporal Gyrus, anterior division**	-0.143	0.122	0.258	0.524	-0.053	0.071	0.487	-0.02	-0.142	-0.202	**0.09**

**Paracingulate Gyrus**	0.052	-0.194	0.217	0.156	0.536	0.311	0.22	-0.34	0.188	-0.385	**0.076**
**Cingulate Gyrus, posterior division**	0.153	-0.177	0.05	0.416	0.473	0.037	0.333	-0.171	-0.295	-0.19	**0.063**
**Middle Temporal Gyrus, anterior division**	0.025	-0.106	0.117	0.037	0.126	0.33	0.36	-0.01	-0.274	-0.012	**0.059**
**Angular Gyrus**	-0.12	-0.225	-0.022	0.551	0.363	-0.026	0.339	-0.199	0.033	-0.232	**0.046**
**Temporal Fusiform Cortex, anterior division**	-0.246	0.116	0.286	0	0.111	0.074	-0.04	0.005	0.163	-0.188	**0.028**
**Superior Parietal Lobule**	-0.077	-0.293	-0.074	-0.008	0.431	0.434	0.294	-0.462	0.058	-0.323	**-0.002**
**Planum Polare**	-0.031	-0.071	-0.111	0.005	-0.058	0.145	-0.006	-0.007	0.114	-0.067	**-0.009**
**Occipital Pole**	-0.026	-0.325	0.147	0.267	0.188	-0.114	0.378	-0.338	-0.238	-0.227	**-0.029**
**Frontal Opercular Cortex**	-0.052	-0.177	-0.163	0.058	0.169	-0.08	0.111	-0.033	0.114	-0.418	**-0.047**
**Parietal Opercular Cortex**	-0.108	-0.479	0.014	0.225	0.236	-0.114	0.163	-0.329	-0.12	-0.268	**-0.078**
**Superior Frontal Gyrus**	0.125	-0.476	-0.165	0.164	0.057	-0.138	0.399	-0.696	-0.385	-0.479	**-0.159**
**Heschl's Gyrus (includes H1 and H2)**	-0.421	-0.476	-0.13	0.077	-0.077	-0.083	-0.391	0.195	-0.143	-0.513	**-0.196**
**Insular Cortex**	-0.148	-0.643	-0.134	-0.026	-0.106	-0.062	-0.245	-0.189	-0.47	-0.047	**-0.207**
**MEAN MODEL**	0.26	0.04	0.188	0.41	0.451	0.291	0.418	-0.024	0.194	0.111	**0.234**

TABLE H: ROI statistics for regression models (RBC values)

The "Mean Region" column summarizes the predictability for a given ROI by averaging the RBC values of the different models. The ROIs are sorted in descending order based on this column, so that the ROIs with the highest general predictability appear first.

	Linear	KNN	DT	RF	SVM	XGB	MLP	CNN	RNN	Transformer	MEAN REGION
**Inferior Temporal Gyrus, posterior division**	-0.222	-0.323	-0.598	0.148	0.344	-0.349	-0.254	0.323	0.085	0.354	**-0.049**
**Cuneal Cortex**	-0.286	0.016	-0.698	0.317	0.64	-0.307	-0.265	-0.265	-0.413	0.296	**-0.096**
**Inferior Frontal Gyrus, pars opercularis**	-0.889	-0.148	-0.704	0.312	0.614	-0.476	0.069	0.455	-0.127	-0.074	**-0.097**
**Frontal Medial Cortex**	-0.646	-0.143	-0.418	0.55	0.333	-0.365	-0.042	-0.206	-0.079	-0.053	**-0.107**
**Intracalcarine Cortex**	-0.312	-0.333	-0.519	0.317	0.545	-0.492	-0.439	-0.037	-0.317	0.45	**-0.114**
**Lateral Occipital Cortex, inferior division**	-0.54	-0.513	-0.624	0.249	0.27	-0.571	-0.312	0.344	0.042	0.312	**-0.134**
**Occipital Fusiform Gyrus**	-0.762	-0.508	-0.709	0.423	0.349	-0.64	-0.048	0.265	-0.18	0.122	**-0.169**
**Middle Temporal Gyrus, posterior division**	-0.524	-0.434	-0.582	-0.048	0.291	-0.545	-0.164	0.302	-0.085	0.095	**-0.169**
**Inferior Temporal Gyrus, temporooccipital part**	-0.45	-0.317	-0.772	0.011	0.333	-0.714	-0.265	0.18	-0.063	0.344	**-0.171**
**Supramarginal Gyrus, anterior division**	-0.968	-0.101	-0.81	0.323	0.614	-0.646	0.021	0.328	-0.429	-0.106	**-0.177**
**Supracalcarine Cortex**	-0.783	-0.069	-0.593	0.376	0.508	-0.529	-0.381	0.116	-0.656	0.063	**-0.195**
**Supramarginal Gyrus, posterior division**	-0.952	-0.185	-0.825	0.365	0.598	-0.725	-0.127	-0.005	-0.127	0.026	**-0.196**
**Lingual Gyrus**	-0.783	-0.476	-0.524	0.497	0.434	-0.513	-0.302	0.032	-0.392	0.026	**-0.2**
**Temporal Occipital Fusiform Cortex**	-0.937	-0.503	-0.688	0.529	0.323	-0.894	-0.212	-0.074	-0.116	0.434	**-0.214**
**Precuneous Cortex**	-0.963	-0.238	-0.746	0.307	0.323	-0.751	0.085	0.037	-0.291	-0.053	**-0.229**

**Middle Temporal Gyrus, temporooccipital part**	-0.825	-0.619	-0.825	0.228	0.376	-0.82	-0.249	0.122	-0.222	0.291	**-0.254**
**Frontal Opercular Cortex**	-0.942	-0.19	-0.402	0.217	0.439	-0.709	-0.307	-0.296	-0.481	-0.169	**-0.284**
**Temporal Pole**	-0.672	-0.27	-0.37	-0.021	0.127	-0.656	-0.397	-0.27	-0.587	-0.048	**-0.316**
**Superior Temporal Gyrus, posterior division**	-0.905	-0.677	-0.804	0.101	0.079	-0.91	-0.016	0.407	-0.27	-0.201	**-0.32**
**Juxtapositional Lobule Cortex (formerly Supplementary Motor Cortex)**	-0.656	-0.196	-0.571	0.148	0.423	-0.503	-0.571	-0.519	-0.746	-0.132	**-0.332**
**Inferior Frontal Gyrus, pars triangularis**	-0.968	-0.397	-0.73	-0.079	0.132	-0.873	-0.201	0.37	-0.063	-0.513	**-0.332**
**Middle Frontal Gyrus**	-0.984	-0.429	-0.672	0.09	0.243	-0.889	-0.069	0.011	-0.54	-0.095	**-0.333**
**Occipital Pole**	-0.878	-0.64	-0.677	0.09	0.053	-0.757	-0.323	0.09	-0.503	0.132	**-0.341**
**Frontal Pole**	-0.968	-0.571	-0.772	-0.074	0.143	-0.894	0.153	0.243	-0.291	-0.466	**-0.35**
**Inferior Temporal Gyrus, anterior division**	-0.836	-0.503	-0.635	-0.011	-0.037	-0.73	0.079	-0.212	-0.503	-0.254	**-0.364**
**Precentral Gyrus**	-0.868	-0.571	-0.693	0.011	0.27	-0.709	-0.407	-0.095	-0.302	-0.323	**-0.369**
**Parietal Opercular Cortex**	-0.937	-0.397	-0.593	0.116	0.048	-0.889	-0.333	0	-0.275	-0.677	**-0.394**
**Parahippocampal Gyrus, posterior division**	-0.81	-0.444	-0.497	-0.132	0.09	-0.804	-0.328	-0.243	-0.704	-0.079	**-0.395**
**Lateral Occipital Cortex, superior division**	-0.937	-0.762	-0.746	0.143	-0.106	-0.937	-0.143	-0.095	-0.571	0.164	**-0.399**
**Subcallosal Cortex**	-0.878	-0.513	-0.497	-0.016	-0.042	-0.831	-0.286	-0.661	-0.413	0.074	**-0.406**
**Temporal Fusiform Cortex, anterior division**	-0.91	-0.476	-0.614	-0.201	-0.058	-0.857	-0.434	-0.143	-0.593	0.222	**-0.406**
**Superior Frontal Gyrus**	-0.841	-0.577	-0.603	-0.026	0.138	-0.751	-0.534	-0.053	-0.524	-0.376	**-0.415**
**Parahippocampal Gyrus, anterior division**	-0.751	-0.455	-0.418	-0.101	-0.079	-0.751	-0.333	-0.407	-0.63	-0.28	**-0.421**
**Angular Gyrus**	-0.979	-0.524	-0.884	0.021	0.016	-0.968	-0.095	-0.011	-0.328	-0.561	**-0.431**
**Central Opercular Cortex**	-0.989	-0.45	-0.608	-0.127	0.127	-0.868	-0.085	-0.175	-0.598	-0.561	**-0.433**
**Postcentral Gyrus**	-0.958	-0.64	-0.656	-0.122	-0.09	-0.836	-0.254	0	-0.608	-0.222	**-0.439**

**Superior Parietal Lobule**	-0.989	-0.296	-0.534	-0.106	0.106	-0.926	-0.418	-0.307	-0.508	-0.444	**-0.442**
**Temporal Fusiform Cortex, posterior division**	-0.899	-0.561	-0.82	-0.138	-0.312	-0.905	-0.365	-0.296	-0.175	-0.111	**-0.458**
**Planum Temporale**	-0.995	-0.566	-0.794	-0.354	0.074	-0.937	-0.381	0.275	-0.635	-0.296	**-0.461**
**Cingulate Gyrus, anterior division**	-0.91	-0.423	-0.481	-0.249	0.116	-0.772	-0.466	-0.497	-0.624	-0.376	**-0.468**
**Superior Temporal Gyrus, anterior division**	-1	-0.741	-0.788	-0.058	-0.079	-0.968	-0.519	0.201	-0.471	-0.291	**-0.471**
**Insular Cortex**	-0.905	-0.429	-0.54	-0.153	0.016	-0.857	-0.317	-0.614	-0.81	-0.143	**-0.475**
**Frontal Orbital Cortex**	-0.91	-0.825	-0.783	0.063	-0.074	-0.836	-0.714	0.09	-0.476	-0.312	**-0.478**
**Cingulate Gyrus, posterior division**	-1	-0.439	-0.587	-0.254	0.042	-0.91	-0.423	-0.323	-0.608	-0.529	**-0.503**
**Middle Temporal Gyrus, anterior division**	-0.952	-0.656	-0.635	-0.021	-0.254	-0.825	-0.619	-0.286	-0.63	-0.243	**-0.512**
**Paracingulate Gyrus**	-1	-0.423	-0.677	-0.138	0.185	-0.889	-0.598	-0.407	-0.46	-0.831	**-0.524**
**Heschl's Gyrus (includes H1 and H2)**	-1	-0.709	-0.534	-0.116	-0.106	-0.963	-0.566	-0.175	-0.55	-0.598	**-0.532**
**Planum Polare**	-0.979	-0.783	-0.386	-0.291	-0.413	-0.952	-0.423	-0.175	-0.608	-0.508	**-0.552**
**MEAN MODEL**	-0.834	-0.446	-0.638	0.065	0.169	-0.754	-0.283	-0.055	-0.405	-0.136	**-0.332**

TABLE I: ROI statistics for foundation models (RBC values), models evaluated on 8 brain regions

These statistics are given for completeness, but should be considered with caution. Not only are the foundation models evaluated on a selection of our fMRI scans of interest, but missing or ambiguous predictions must be dynamically excluded, resulting in significantly fewer data points. Region-wise analyses may not be as reliable as for the classical machine learning and deep learning models.

	Gemma	Llama	PaliGemma	Gemma 5-Channel	MEAN REGION
**Frontal Pole**	0.467	0.289	-0.733	0.244	**0.067**
**Superior Parietal Lobule**	0.378	0.156	-0.467	-0.2	**-0.033**
**Insular Cortex**	-0.022	-0.378	0.333	-0.467	**-0.133**
**Occipital Pole**	0.6	-0.244	-0.511	-0.422	**-0.144**
**Angular Gyrus**	0.067	0.156	-0.644	-0.333	**-0.189**
**Inferior Frontal Gyrus, pars triangularis**	-0.111	0.067	-0.6	-0.378	**-0.256**
**Superior Temporal Gyrus, posterior division**	0.022	-0.333	-0.733	-0.244	**-0.322**
**Precentral Gyrus**	-0.244	-0.333	-0.6	-0.244	**-0.356**
**MEAN MODEL**	0.144	-0.078	-0.494	-0.256	**-0.171**

TABLE J: ROI statistics for foundation models (RBC values), models evaluated on 5 brain regions

These statistics are given for completeness, but should be considered with caution. Not only are the foundation models evaluated on a selection of our fMRI scans of interest, but missing or ambiguous predictions must be dynamically excluded, resulting in significantly fewer data points. Region-wise analyses may not be as reliable as for the classical machine learning and deep learning models.

	Gemma CoT	Gemma without FT	Gemma with FT	MEAN REGION
**Precentral Gyrus**	0.644	0.467	0.422	**0.511**
**Superior Parietal Lobule**	-0.156	0.289	0.556	**0.23**
**Occipital Pole**	0.289	-0.111	0.156	**0.111**
**Superior Temporal Gyrus, posterior division**	0.022	0.156	0.111	**0.096**
**Frontal Pole**	-0.067	0.2	-0.378	**-0.081**
**MEAN MODEL**	0.147	0.2	0.173	**0.173**
